# The Genetic Landscape of Androgenetic Alopecia: Current Knowledge and Future Perspectives

**DOI:** 10.3390/biology15020192

**Published:** 2026-01-21

**Authors:** Aditya K. Gupta, Daniel J. Dennis, Vasiliki Economopoulos, Vincent Piguet

**Affiliations:** 1 Mediprobe Research Inc., London, ON N5X 2P1, Canada; ddennis@mediproberesearch.com (D.J.D.); veconomopoulos@mediproberesearch.com (V.E.); 2 Division of Dermatology, Department of Medicine, Temerty Faculty of Medicine, University of Toronto, Toronto, ON M5S 3H2, Canada; vincent.piguet@utoronto.ca; 3 Division of Dermatology, Department of Medicine, Women’s College Hospital, Toronto, ON M5S 1B2, Canada

**Keywords:** androgenetic alopecia, male-pattern hair loss, female-pattern hair loss, genome wide association studies

## Abstract

Androgenetic alopecia (AGA) is the most common cause of progressive hair thinning in adults and has traditionally been viewed as an androgen-driven inherited condition. Genomic research now demonstrates that AGA is a complex polygenic disorder involving multiple biological pathways, including androgen signaling, hair follicle development, cell survival, and extracellular matrix remodeling. Genome-wide association studies have identified numerous susceptibility loci, revealing that follicle miniaturization arises from interacting molecular mechanisms rather than a single pathogenic process. Genetic risk and predictive value vary across populations, with many loci identified in European cohorts showing limited transferability to other ancestries, highlighting the need for more diverse genetic studies. In women, genetic studies remain underpowered, and emerging data suggest partially distinct risk architecture compared with male AGA. Pharmacogenetic findings indicate that genetic variation may influence response to commonly used therapies, although no markers are currently validated for routine clinical use. Advances in single-cell and multi-omic approaches are improving understanding of how genetic risk translates into follicular dysfunction, supporting the development of more personalized and mechanism-based treatment strategies.

## Simple Summary

Androgenetic hair loss is the most common cause of gradual hair thinning in adults. For many years, this form of hair loss was thought to be driven mainly by male hormones and inherited in a simple way. Recent genetic research has shown that hair loss is influenced by many genes that affect how hair follicles grow, survive, and respond to hormones. These genes act through several biological pathways, leading to progressive thinning of hair follicles over time. Genetic risk also differs between populations, meaning that results from one ancestry group may not apply equally to others. In women, the genetic factors involved in hair loss appear partly different from those in men, and more research focused on women is still needed. Studies are also beginning to show that a person’s genetic makeup may influence how well certain hair loss treatments work or whether side effects might occur. New methods that study individual cells in hair follicles are improving understanding of how genetic risk leads to hair thinning and may help guide more personalized treatments in the future.

## 1. Introduction

Androgenetic alopecia (AGA), commonly known as male-pattern or female-pattern hair loss, is the most prevalent form of progressive hair thinning in adults. Epidemiological studies estimate that nearly 70% of Caucasian men will experience some degree of AGA by age 70 years, and nearly 50% of women [1,2,3]. Men typically have an earlier onset of hair loss than women [4]. The condition is characterized histologically by the miniaturization of terminal hair follicles and a shortening of the anagen (growth) phase [5,6], driven by androgen signaling in genetically susceptible hair follicles [7]. Genetically, AGA is among the most heritable dermatological traits: twin and familial studies have suggested heritability estimates up to ~80%, pointing to a strong polygenic architecture [8,9].

However, early candidate-gene association studies, most notably involving the androgen receptor (AR) locus on the X chromosome and the 20p11 region, accounted for only a fraction of the inherited risk [10], highlighting the need for more comprehensive genome-wide efforts.

In recent years, large-scale genome-wide association studies (GWAS) have dramatically expanded our understanding of AGA’s genetic architecture. A large GWAS identified 71 AGA risk loci, and highlighted genes across multiple biological pathways, such as WNT signaling [11].

Similarly, a key meta-analysis implicated biological pathways such as melatonin signaling and adipogenesis [12]. Despite this progress, much of the heritable risk for AGA remains unexplained, and the discovery of risk alleles has been uneven across populations. For instance, studies in populations of non-European ancestries have validated some established loci while also identifying novel variants [13,14,15], underscoring the importance of ancestry-specific studies. Given these advances, there is a critical need to translate genetic findings into biological mechanisms, clinical risk prediction, and ultimately therapeutic strategies. Functional characterization of GWAS loci, integration with multi-omics and single-cell data, and pharmacogenetic insights are increasingly informing our understanding of how genetic variation shapes both disease susceptibility and treatment response. In this review, we synthesize the current literature on AGA genetics, with a focus on genome-wide risk loci, mechanistic pathways, sex and ancestry differences, and the emerging clinical implications for pharmacogenetics.

## 2. Genetic Architecture of AGA

### 2.1. Genome-Wide Association Studies

Over the past decade, genome-wide association studies have defined AGA as a highly polygenic trait with dozens of loci of small to moderate effect. Early studies built on classic candidate gene findings at the androgen receptor (AR) locus and the 20p11 region, but expanded the architecture substantially [16,17].

A landmark GWAS in over 70,000 men of European ancestry identified 71 independent susceptibility loci, explaining approximately 38% of the single nucleotide polymorphism (SNP)-based risk for AGA, and implicating genes across biologically coherent pathways including WNT signaling, androgen metabolism, apoptosis, and morphogenesis [11]. This built on an earlier meta-analysis that had expanded candidate loci to implicate genes such as *FGF5*, *IRF4*, *DKK2*, and pathways including melatonin signaling and adipogenesis [12]. Because many participants in these studies were aged 50 years and older, it is plausible that some of the non-androgenic loci (e.g., WNT-pathway) reflect a mixture of classical AGA and age-related (senescent) hair thinning, rather than AGA alone [18].

While functional analysis has not been performed on most SNPs, more recent post-GWAS approaches, including transcriptome-wide association studies (TWAS) have begun to identify more functional gene-level candidates [19]. The study identified several novel genes, including *CD59* (cell survival and apoptosis), *ZDHHC5* (membrane localization), and *ZIC2* (follicle morphogenesis). While most of the work on identifying SNPs has been in individuals with European ancestry, recent GWAS have used subjects with non-European ancestry (discussed in under Ancestry considerations). Although genetic studies of AGA have largely focused on common variants identified by genome-wide association studies, analysis of recent large-scale exome sequencing data from the UK Biobank suggests that rare genetic variants play a relatively small role in AGA risk, with associations limited to few (5) genes, two of which (*EDA2R* and *WNT10A*) are already implicated by GWAS, rather than evidence for rare, high-impact mutations [20].

### 2.2. Key Biological Pathways

**Figure 1:**
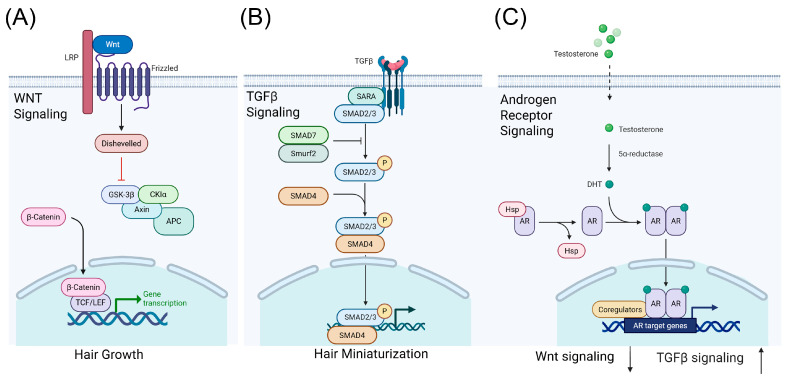
Key signaling pathways involved in the regulation of hair follicle growth and androgenetic alopecia (AGA). (**A**) Wnt signaling promotes hair follicle proliferation and maintenance, supporting normal hair growth. (**B**) TGF-β signaling induces hair follicle miniaturization, contributing to the progressive thinning of hair observed in AGA. (**C**) Androgen receptor (AR) signaling mediates the effects of androgens on hair follicles, resulting in decreased Wnt signaling and increased TGF-β signaling, which together drive hair follicle miniaturization and hair loss. GSK-3β—Glycogen synthase kinase-3 beta; APC—Adenomatous polyposis coli; CKIα—Casein kinase I alpha; TCF/LEF—T-cell factor/lymphoid enhancer factor; SARA—Smad anchor for receptor activation; SMAD—Suppressor of mothers against decapentaplegic; P—phosphorylation; HSP—Heat shock protein; AR—Androgen receptor; DHT—Dihydrotestosterone.

Although GWAS loci are dispersed across the genome, functional annotation and follow-up studies repeatedly implicate a limited number of coherent biological pathways. Prominent among these are androgen metabolism/AR-mediated signaling [21,22,23], WNT/β-catenin signaling (for example WNT10A and its regulatory partners) [24], pathways controlling hair-follicle morphogenesis and stem/progenitor cell function [25], and pathways related to apoptosis, extracellular matrix remodeling, and energy metabolism (Table 1) [25,26]. Expression studies in human scalp follicles from AGA patients reveal altered regulation of extracellular matrix and progenitor cell genes, suppression of canonical WNT/β-catenin signaling, increases in TGF-β signaling (which can lead to hair miniaturization through premature transition to the catagen phase) [27], and dysregulated structural and metabolic pathways in miniaturizing follicles (Figure 1) [25,26]. Together, these findings support a model in which genetic variation influences follicle regeneration and susceptibility to miniaturization in androgenetic alopecia [21,22].

**Table 1:** Genes and associated SNPs implicated in AGA.

Gene/Locus	SNP or Variant	Population	Risk
*AR* [23]	rs6152	European mixed ancestry male populations	Increased risk
*AR/EDA2R* locus [23,24,28,29]	rs12558842	European mixed ancestry male population; Mixed ethnicity mixed sex; German male population	Increased risk
Xq12 locus [30]	rs1041668	European mixed ancestry male population	Increased risk
20p11 locus [30]	rs1160312	European mixed ancestry male population	Increased risk
	rs6113491	European mixed ancestry male population	Increased risk
*WNT10A* intronic region [24]	rs7349332	European mixed ancestry male population	Increased risk
*SUCNR1* and *MBNL1* intergenic [24]	rs7648585	European mixed ancestry male population	Increased risk
*EBF1* [23,24]	rs929626	European mixed ancestry male population	Reduced risk [22,29]
	rs1081073	European mixed ancestry male population	Increased risk [29]
*SSPN* and *ITPR2* intergenic [24]	rs9668810	European mixed ancestry male population	Increased risk
	rs7975017	European mixed ancestry male population	Increased risk
*HDAC9* [28,31]	rs2073963	German male population; European mixed ancestry mixed sex population	Increased risk
*TARDBP* [23,31]	rs12565727	European mixed ancestry male population; European mixed ancestry mixed sex population	Reduced risk [22]; Increased risk [30]
*AUTS2* [31]	rs6945541	European mixed ancestry mixed sex population	Increased risk
*PAX1* and *FOXA2* intergenic [31,32]	rs6047844	European mixed ancestry mixed sex population, Korean males	Increased risk
*PTGES2* [33]	rs13283456	Mixed ethnicity and mixed sex population	Increased risk
*SRD5A2* [33]	rs523349	Mixed ethnicity and mixed sex population	Increased risk
*COL1A1* [33]	rs1800012	Mixed ethnicity and mixed sex population	Increased risk
*ACE* [33]	rs4343	Mixed ethnicity and mixed sex population	Increased risk
*PTGFR* [33]	rs10782665	Mixed ethnicity and mixed sex population	Increased risk
*PTGDR2* [33]	rs533116	Mixed male and female population	Increased risk
	rs545659	Mixed ethnicity and mixed sex population	Increased risk
*CRABP2* [33]	rs12724719	Mixed ethnicity and mixed sex population	Increased risk
Not known [13]	rs11010734	Korean male population	Increased risk
*PANK1* and *KIF20B* intergenic [13]	rs2420640	Korean male population	Increased risk
2q31.1 locus [14]	rs13405699	Han Chinese male population	Increased risk
*FGF5* [12]	rs982804	European mixed ancestry male population	Increased risk
*IRF4* [12]	rs12203592	European mixed ancestry male population	Increased risk
*DKK2* [12,15]	rs145945174	European mixed ancestry male population	Increased risk [12]
	rs116494345	African male population	Increased risk [15]
*SLC301A10* [15]	rs143451223	African male population	Increased risk
*FZD1* [32]	rs2163085	Korean female population	Increased risk
*GJC1* [32]	rs4793158	Korean female population	Increased risk

### 2.3. Sex Differences

Sex modifies both epidemiology and genetic architecture in patterned hair loss [34]. Classic candidate-gene work and later genomic studies show a major contribution from variants at the AR locus on the X chromosome, explaining part of the strong male bias in many cohorts [2]. However, when known male AGA risk loci have been tested in women with female-pattern hair loss (FPHL), results have been largely negative or inconclusive [32,35]. This may reflect not only true biological differences between male- and female-patterned hair loss, but also substantial phenotypic heterogeneity and diagnostic ambiguity within FPHL cohorts. In clinical practice and epidemiologic studies, diffuse hair thinning in women is frequently classified as FPHL despite the possibility that multiple biological processes, such as chronic telogen effluvium, age-related (senescent) alopecia, or mixed phenotypes, may contribute to the observed pattern [36,37]. Such phenotypic misclassification is likely to dilute genetic signals and reduce power to detect reproducible susceptibility loci.

Early attempts to replicate male AGA loci in women found no significant association at 20p11 and only a nominal *AR*/*EDA2R* signal, with subsequent analysis of additional male loci failing to replicate in FPHL cohorts [35,38]. Although these findings raised the question of whether FPHL might instead reflect senescence-related hair thinning, current evidence suggests that age-related changes alone cannot fully account for the phenotype [39]. As in male cohorts enriched for older individuals, where classical AGA may coexist with senescent alopecia, genetic studies in women are similarly vulnerable to phenotypic admixture. Diffuse thinning in women may reflect a mixture of androgen-dependent FPHL, age-related hair thinning, and non-androgenetic processes, which can obscure true genetic associations and contribute to inconsistent replication across studies [36,39].

More recent work supports the presence of female-specific contributions [32]. In a sex-stratified genome-wide association study of a Korean population, several loci reached significance exclusively in women, such as rs2163085 (*FZD1*) and rs4793158 (*GJC1*) [32]. These findings provide some of the first genome-wide evidence that the genetic determinants of FPHL are at least partially distinct from those underlying male-pattern hair loss. However, the current literature remains limited by small sample sizes, heterogeneous case definitions, and inconsistent phenotyping, making it difficult to disentangle true sex-specific genetic architecture from methodological confounding. Extrapolating risk models from male-derived GWAS to women due to a lack in female-only studies is likely insufficient, highlighting the need for well-powered female-specific GWAS.

### 2.4. Ancestry Considerations

Most published discovery GWAS have used European-ancestry cohorts, which has two consequences: (1) many discovered loci have been optimized to European linkage disequilibrium (LD) patterns and allele frequencies, meaning that the lead SNPs identified in these studies are the variants that best tag the underlying causal variants in European populations but may not tag them well in other ancestries, and (2) polygenic risk scores trained in Europeans attenuate when applied to non-European populations. In European populations, AR variants have been linked not only to AGA risk but also to earlier age of onset [22], providing a direct connection between genetic architecture and observed population-level clinical features. These issues have started to be addressed by a recent shift in the field toward ancestry-diverse studies.

Independent studies in East Asian and African populations have both replicated several canonical loci, such as 20p11 and 2q31.1, and identified additional, population-specific signals [13,15,32,40], underscoring the need for broader ancestral representation across studies of varying design and statistical power (Table 2).

Recent work provides some of the first broad replication and pilot genome-wide evidence for AGA risk in Asian populations. A Korean study confirmed that SNPs in the 20p11 region are associated with AGA, consistent with findings from European cohorts, but variants in *AR* and *EDA2R* were not associated in Korean individuals [13]. Importantly, the study identified two SNPs, rs11010734 and rs2420640, found at 10p11.21 and near *PANK1*-*KIF20B*, respectively, that were uniquely associated with AGA risk in Korean populations [13]. Much like Korean populations, the 20p11 region has been previously associated with AGA risk in Chinese populations [41]. In addition, Chinese populations had shown an association with the 2q31.1 SNP rs13405699 and several other genes, including *WNT10A* (rs7349332), that had previously been reported in populations with European ancestry [14]. Population-level observations suggest that AGA tends to present with later onset and more diffuse hair thinning in East Asian men compared with Europeans [42], although the specific contribution of these SNPs to these clinical differences remains to be formally established.

Very recent work in African men by Janivara et al. found that European-derived polygenic risk scores for AGA performed poorly in African men, showing minimal predictive ability [15]. The authors identified several loci associated with AGA, including 36 associations that appear unique to African populations, including rs116494345 at 1p13.2 and rs143451223 at 1q41 [15]. The strongest signal was at 1p13.2, where the lead SNP rs116494345 is monomorphic in European and Asian populations, representing a truly African-specific association. Most AGA-associated variants in this study were autosomal, and unlike findings in European cohorts, the X chromosome did not show strong associations with AGA in African men. Epidemiological studies indicate that African men generally exhibit lower overall prevalence of AGA [43], consistent with the possibility of population-specific genetic effects.

Taken together, these findings highlight that the genetic architecture of AGA is partially population-specific. This has important implications, as polygenic risk scores or therapeutic predictions derived from European cohorts may not generalize to other ancestries, and novel, ancestry-specific loci may reveal new biological pathways or targets for intervention.

**Table 2:** Evidence strength from key studies.

Author	Select Genes/Loci	Study Type	Sample Size (Cases/Total N)	Strength of Evidence
Ambra et al. 2025 [30]	Xq12 locus, 20p11 locus	Genetic association study	104 cases/ 212 N	Moderate—single study with a relatively small sample, associations adjusted for confounders, but no replication cohort or meta-analysis included
Brockschmidt et al. 2011 [28]	*AR/EDA2R* locus, *HDAC9*	GWAS	581 cases/ 1198 N	Moderate to strong—replicated in independent sample; supported by fine-mapping, family-based TDT analysis, and tissue expression studies; effect sizes modest
Francès et al. 2024 [33]	*PTGES2*, *SRD5A2*, *COL1A1*, *ACE*, *PTGFR*, *PTGDR2*, *CRABP2*	Candidate SNP association study	26,607/ 26,607 N	Low to moderate—Large sample size improves statistical power, However, restricted to predefined candidate SNPs. Associations reported at nominal significance thresholds (*p* < 0.05), not genome-wide significance. Absence of non-AGA controls limits inference about disease susceptibility
Heilmann et al. 2013 [24]	*WNT10A*, *SUCNR1* and *MBNL1* intergenic, *EBF1*, *SSPN* and *ITPR2* intergenic	Replication of meta-analysis	2759/5420 N (plus previous meta-analysis)	Strong—genome-wide significant loci confirmed, multi-cohort replication, robust QC and statistical methods, supported by expression analysis in human hair follicles
Heilmann-Heimbach et al. 2017 [12]	*FGF5*, *IRF4*, *DKK2*	GWAS	10,846/ 26,607 N	Very strong—high-quality genetic evidence. Genome-wide significance threshold applied (*p* < 5 × 10^−8^) Extensive quality control, imputation, and heterogeneity testing. Replication across multiple independent cohorts. Polygenicity formally assessed and population stratification ruled out. Functional follow-up (eQTLs, enhancer enrichment, pathway analyses)
Henne et al. 2023 [44]	*EDA2R*, *WNT10A*	Exome wide association	72,469/ 72,469 N	Strong—very large, population-based exome sequencing study; combines single-variant and gene-based tests; confirms known genes and identifies novel rare variant associations; results are further integrated with PRS for risk modeling
Janivara et al. 2025 [15]	*DKK2*, *SLC301A10*	GWAS	2136/ 2136 N	Moderate—limited by moderate sample size for detecting genome-wide significance and by reliance on self-reported baldness
Kim et al. 2022 [13]	*PANK1* and *KIF20B* intergenic	GWAS	275/421 N	Low to moderate—Single-center, hospital-based cohort, modest sample size with limited statistical power, GWAS findings reach suggestive significance rather than conventional genome-wide significance, replication signals largely nominal (*p* < 0.05)
Lee et al. 2024 [32]	*PAX1* and *FOXA2* intergenic, *FZD1*, *GJC1*	GWAS	545/1004 N	Moderate—Single-cohort GWAS with modest sample size, includes replication of known loci, limited power relative to large meta-analyses
Li et al. 2012 [31]	*HDAC9*, *TARDBP*, *AUTS2*, *PAX1* and *FOXA2* intergenic	Meta-analysis of GWAS	3891/ 12,806 N	Strong—Large-scale GWAS meta-analysis across multiple cohorts, genome-wide significant loci identified (*p* < 5 × 10^−8^), replication and follow-up in independent samples, multiple analyses including risk score and disease association
Li et al. 2024 [14]	2q31.1	Candidate SNP replication	499/1988 N	Moderate—Well-powered replication of known GWAS loci, but only 1 SNP reached significance after multiple testing, limited by relatively small sample size
Marcińska et al. 2015 [23]	*AR*, *AR/EDA2R* locus, *EBF1*, *TARDBP*	Candidate SNP association study	476/605 N	Moderate—validated in independent test set, moderate sample size
Zhuo et al. 2012 [29]	*AR/EDA2R* locus	Meta-analysis	2074/3189 N	Moderate—synthesizes multiple studies, limited by small number of included studies (*n* = 8) and some heterogeneity

## 3. Genetic Associations and Biological Mechanisms

### 3.1. Functional Annotation of GWAS Loci

GWAS have repeatedly implicated the AR locus and an androgen-independent signal at 20p11 as major AGA susceptibility regions [11]. Translating locus associations to genes requires ancestry-aware fine-mapping, integration with expression quantitative trait loci (eQTL) and chromatin annotations, and colocalization to prioritize candidate causal genes and regulatory elements [45,46]. Such functional annotations have emphasized that many lead SNPs are tag variants and that differing linkage disequilibrium and allele frequencies between ancestries change which variants best tag a locus [47].

### 3.2. Insights from Single-Cell and Multi-Omics Studies

Single-cell and spatial transcriptomic work on human scalp and follicles is beginning to map cell types and regulatory states relevant to hair cycling [48,49,50]. Single-cell atlases of human scalp hair follicles provide lineage maps that can help localize GWAS-nominated genes to specific follicular cell compartments.

While AGA-specific single-cell studies remain limited, recent work from Ober-Reynolds et al. have utilized this approach to link AGA GWAS loci with regulatory elements in scalp tissue [51]. Notably, they observed strong enrichment of AGA-associated SNPs within open chromatin regions in dermal papilla cells, implicating this mesenchymal population as a key mediator of genetic risk [51]. They further predicted that *WNT10A* SNP rs72966077 was functionally important in AGA, providing mechanistic insight into how at least some risk loci may influence hair-follicle biology.

Single-cell studies of human scalp follicles further resolve epithelial and progenitor populations, enabling future integration of transcriptomic data to determine cell-type-specific mechanisms in AGA [45,46]. Together, these findings suggest that single-cell and chromatin mapping can link GWAS loci to candidate genes and regulatory networks, generating hypotheses for how biological pathways contribute to AGA.

### 3.3. Clinical Significance of Biological Mechanisms

The strongest translationally actionable mechanism for AGA remains androgen signaling, with AR and local dihydrotestosterone (DHT) biology rationalizing 5-alpha reductase inhibitors and AR-targeted approaches for many patients [45]. However, loci such as 20p11 and multiple other autosomal GWAS hits implicate androgen-independent pathways (e.g., WNT/TGF-β) that likely contribute to variable treatment response and offer alternative therapeutic targets [11,12,44]. The WNT signaling pathway is important for regulating hair follicle regeneration [52]. A *WNT10A* polymorphism (rs7349332) is strongly associated with AGA and may result in lower *WNT10A* expression [14,24]. Topical tretinoin may be a potential treatment to target the WNT pathway, as improved hair growth was observed in a recent clinical study [53].

## 4. Pharmacogenetics and Clinical Implications

### 4.1. Finasteride and Dutasteride

Finasteride and dutasteride reduce DHT by inhibiting type II and type I/II 5-α-reductase, respectively, and are the most effective systemic therapies for AGA in men [54]. Although genetic predictors of treatment response have been proposed, no validated pharmacogenetic markers currently guide finasteride or dutasteride use in AGA.

Studies of *SRD5A2* variants have shown functional effects in prostate disorders but have not demonstrated consistent associations with clinical response in AGA [40,55]. Overall, pharmacogenetic stratification for 5-α-reductase inhibitors remains investigational, and therapeutic decisions rely on clinical factors rather than genotype. Larger studies are still needed before genetic testing can predict finasteride and dutasteride outcomes.

In terms of treatment, hormonal therapies such as finasteride and dutasteride are effective in men with AGA [56], but clinical trials in women show variable efficacy, reflecting both hormonal differences and the distinct genetic architecture of FPHL [57]. While functional analysis has not been performed on most SNPs, a more recent transcriptome-wide association study (TWAS) was performed to identify more functional gene-level candidates [19]. The study identified several novel genes, including *CD59* (cell survival and apoptosis), *ZDHHC5* (membrane localization), and *ZIC2* (follicle morphogenesis). While most of the work on identifying SNPs has been in individuals with European ancestry, recent GWAS have used subjects with non-European ancestry (discussed in under Ancestry considerations).

### 4.2. Minoxidil

Topical minoxidil requires activation to minoxidil sulfate by sulfotransferase SULT1A1 [58]. Early work demonstrated that low SULT1A1 activity in plucked hair follicle samples predicts poor response to topical minoxidil [59]. However, a recent study of AGA and oral minoxidil found low SULT1A1 correlated with better response [60], indicating that the relationship between SULT1A1 and minoxidil may depend on the route of administration or other modifiers. Recent genetic data from a 26-SNP panel study found that *SULT1A1* variant rs1042028 was a predictor of poor minoxidil response, suggesting a role for inherited variation in drug responsiveness [61]. Overall, while *SULT1A1* remains a good candidate pharmacogenetic marker for minoxidil response, further large-scale controlled studies are required before routine clinical implementation.

### 4.3. Genetics and Adverse Effects

Reports of persistent sexual or neuropsychiatric symptoms after finasteride remain rare and causality is unconfirmed. Presently, adverse events have not been robustly linked to reproducible genetic polymorphisms across populations. However, early pharmacogenetic studies reported that repeat-length polymorphisms in the *AR* gene, rs4045402 and rs3138869, were more frequent among AGA patients reporting persistent side-effects after finasteride (post-finasteride syndrome) compared with controls [62,63]. For minoxidil, no convincing pharmacogenetic safety markers have been described to date. *CYP3A5* SNPs are biologically plausible candidates for altered finasteride metabolism, as homozygous carriers of rs776746 have lower finasteride clearance in the liver [56]. One small pilot genotyping study did include the *CYP3A5* variant rs776746 (a candidate for drug metabolism), but it did not demonstrate clear associations with adverse effects or drug clearance in that cohort [64].

### 4.4. Other Therapeutic Approaches and Pharmacogenetic Considerations

Several additional therapies are used or are under investigation for androgenetic alopecia, although pharmacogenetic evidence for these approaches is limited. Topical anti-androgens (e.g., spironolactone and flutamide) have shown modest efficacy, particularly in women, but no reproducible genetic predictors of response or safety have been identified [65,66,67].

Low-level light therapy and platelet-rich plasma have demonstrated variable clinical benefit in controlled studies; however, inter-individual differences in response have not been linked to specific genetic variants [68,69]. Other emerging approaches, including botulinum toxin, and agents targeting WNT signaling, remain investigational, and pharmacogenetic data are currently lacking [52,70]. Recent evidence suggests that tretinoin may represent a novel therapeutic approach [53]. Experimental studies show that topical tretinoin promotes hair follicle stem cell activation, accelerates the transition from telogen to anagen, and stimulates hair growth via Wnt/β-catenin pathways [53]. Early clinical data indicate that tretinoin can improve hair counts and density in AGA patients [53]. However, pharmacogenetic predictors of response to RA therapy have not yet been identified.

Overall, outside of androgen suppression and minoxidil activation, genetic modifiers of treatment response for alternative AGA therapies remain largely unexplored, highlighting an unmet need for genotype-stratified clinical studies.

## 5. Future Directions

### 5.1. Research Needs

Moving GWAS findings toward clinical application will require ancestry-aware fine-mapping and functional follow-up to nominate causal variants and cell types, because LD differences mean that GWAS lead variants often do not mark the same causal allele across ancestries. Large, diverse cohorts plus integrated fine-mapping using chromatin annotations and eQTL/colocalization will reduce false leads from tag SNPs and LD differences across populations. Single-cell and spatial multi-omic atlases of human scalp follicles must be expanded and linked to genotypes so that noncoding GWAS signals can be assigned to precise regulatory elements and cell types.

Recent work demonstrating the power of integrated single-cell chromatin + transcriptome maps in skin and hair tissues exemplifies this approach [51]. Although spatial ATAC-seq and related spatial epigenomic methods are still emerging, applying them to scalp tissue would be especially informative for AGA, where many genetic associations are noncoding and likely act through regulatory elements that require in-tissue context to interpret. In parallel, the field needs well-powered pharmacogenetic studies (genotyped clinical cohorts) that prospectively measure treatment response and adverse events for finasteride/dutasteride and minoxidil, including mechanistic biomarkers (e.g., follicular SULT1A1 activity for minoxidil activation). Small trials and biomarker studies are encouraging but underpowered; larger, standardized collections are essential.

Beyond medical therapy, genetic influences on surgical interventions such as hair transplantation remain poorly characterized. Although the principle of donor dominance explains why transplanted occipital follicles, genetically resistant to androgen-mediated miniaturization, generally maintain growth [71], no specific genetic variants or polygenic risk models are currently available to predict graft survival, cosmetic outcome, or long-term success in individual patients. At present, surgical technique and ongoing progression of native hair loss remain the primary determinants of clinical outcome [71], with genetic predictors representing a future research direction.

An additional priority is improved phenotypic definition in genetic studies of patterned hair loss, particularly in women. Standardized diagnostic criteria that distinguish FPHL from chronic telogen effluvium, senescent alopecia, and mixed phenotypes will be essential to reduce misclassification and enhance statistical power. Incorporating clinical patterning, trichoscopic features, hormonal context, and age stratification into GWAS design may clarify true sex-specific genetic architecture and improve reproducibility of susceptibility loci.

### 5.2. Prospective Studies

Prospective, genotype-stratified clinical trials should be prioritized. Examples of high-value designs include (a) randomized trials of topical/oral minoxidil with pre-treatment measurement of follicular SULT1A1 activity or *SULT1A1* genotype to validate predictive value, and (b) trials testing 5-α-reductase inhibitors where SRD5A and AR variants are prespecified moderators of efficacy and safety.

Pilot trials that used SULT1A1 boosters [72] or measured enzyme activity [59,73] show proof of principle but need replication in larger, multi-center cohorts with standardized endpoints. Prospective pharmaco-safety registries that link longitudinal adverse-event reporting with genotype data are also needed to evaluate putative rare, persistent side effects of finasteride and to search for genetic risk markers in an unbiased way. Such registries should use harmonized case definitions and include neuropsychiatric and sexual health outcomes.

### 5.3. Therapeutic Potential

Integrative genomics and multi-omics will reveal new druggable pathways beyond androgen signaling, for example, WNT-modulation, TGF-β axis interventions, and paracrine factors from dermal papilla, which are now being explored in preclinical and early clinical work [74,75]. Reviews of recent drug development efforts highlight small molecules, biologics, and cell-based approaches that could be prioritized by genetics and cell-type evidence.

Near-term translational opportunities include using SULT1A1 activity/genotype to personalize minoxidil prescribing [76] and testing topical finasteride/minoxidil combinations or local AR modulators to minimize systemic exposure [77,78,79]. In the longer term, genotype-informed selection of patients for WNT or stem-cell–directed therapies could improve response and reduce off-target effects; however, rigorous causal validation is required before clinical deployment.

## 6. Conclusions

An increased understanding of the genetics underlying androgenetic alopecia has shifted the view of AGA from the classic, highly heritable, androgen-driven, monogenic trait to a polygenic condition with numerous risk loci converging on multiple biological pathways, including androgen signaling, WNT/TGF-β pathways, apoptosis, and follicular morphogenesis.

Emerging data also indicate that the genetic architecture of female-pattern hair loss differs from that of men, yet female-focused GWAS remain limited. Sex and ancestry influence both susceptibility and the predictive value of genetic variants, underscoring the need for ancestrally diverse, sex-specific studies.

Although GWAS have identified many associations, much of the heritable risk remains unexplained, and functional characterization of causal variants is limited. Emerging single-cell and spatial multi-omic approaches offer the potential to map noncoding variants to specific follicular cell types and regulatory elements. Pharmacogenetic evidence suggests that variation in genes such as *SULT1A1*, *CYP3A5*, *AR*, and *SRD5A2* may affect treatment response and safety, but validated markers for clinical use are not yet available.

Future research should prioritize ancestry-aware fine-mapping, integrative functional studies, and prospective, genotype-stratified clinical trials. Near-term applications include using SULT1A1 activity to personalize minoxidil therapy and refining local modulation of androgen signaling, while longer-term opportunities may involve targeting WNT/TGF-β pathways and stem/progenitor cell populations. Integrating genomic, transcriptomic, and pharmacogenetic data will be essential to realize precision, mechanism-driven management of AGA.

## 7. Methods

A narrative literature review was conducted using PubMed. PubMed searches combined terms for androgenetic alopecia (“androgenetic alopecia,” “male pattern hair loss,” “female pattern hair loss”) with genetic and pharmacogenetic keywords (“genetics,” “GWAS,” “exome sequencing,” “rare variants,” “polygenic risk score”) and clinical outcomes (“pharmacogenetics,” “hair transplantation,” “clinical outcomes”). Searches included publications from 2000 to 2025, limited to studies in humans and published in English. Additional references were identified from bibliographies of relevant articles. No formal systematic review or meta-analysis protocol was followed.

## Data Availability

There are no available data to share as no new data were generated for this work.

## Abbreviations

The following abbreviations are used in this manuscript:

AGA	Androgenetic alopecia
APC	Adenomatous polyposis coli
AR	Androgen receptor
CKIα	Casein kinase I alpha
DHT	Dihydrotestosterone
DKK2	Dickkopf-related protein 2
eQTL	Expression quantitative trait loci
FGF	Fibroblast growth factor
FPHL	Female-pattern hair loss
GSK-3β	Glycogen synthase kinase-3 beta
GWAS	Genome-wide association studies
HSP	Heat shock protein
P	Phosphorylation
SARA	Smad anchor for receptor activation
SMAD	Suppressor of mothers against decapentaplegic
SNP	Single nucleotide polymorphism
TCF/LEF	T-cell factor/lymphoid enhancer factor
TGF-β	Transforming Growth Factor-beta
TWAS	Transcriptome-wide association study

## References

[1] Devjani S., Ezemma O., Kelley K.J., Stratton E., Senna M. (2023). Androgenetic Alopecia: Therapy Update. Drugs.

[2] Lolli F., Pallotti F., Rossi A., Fortuna M.C., Caro G., Lenzi A., Sansone A., Lombardo F. (2017). Androgenetic Alopecia: A Review. Endocrine.

[3] Martinez-Jacobo L., Villarreal-Villarreal C.D., Ortiz-López R., Ocampo-Candiani J., Rojas-Martínez A. (2018). Genetic and Molecular Aspects of Androgenetic Alopecia. Indian J. Dermatol. Venereol. Leprol..

[4] Gupta A.K., Wang T., Economopoulos V. (2025). Epidemiological Landscape of Androgenetic Alopecia in the US: An All of Us Cross-Sectional Study. PLoS ONE.

[5] Paus R., Cotsarelis G. (1999). The Biology of Hair Follicles. N. Engl. J. Med..

[6] Pierard-Franchimont C., Piérard G.E. (2001). Teloptosis, a Turning Point in Hair Shedding Biorhythms. Dermatology.

[7] Inui S., Itami S. (2011). Molecular Basis of Androgenetic Alopecia: From Androgen to Paracrine Mediators through Dermal Papilla. J. Dermatol. Sci..

[8] Nyholt D.R., Gillespie N.A., Heath A.C., Martin N.G. (2003). Genetic Basis of Male Pattern Baldness. J. Investig. Dermatol..

[9] Rexbye H., Petersen I., Iachina M., Mortensen J., Mcgue M., Vaupel J.W., Christensen K. (2005). Hair Loss Among Elderly Men: Etiology and Impact on Perceived Age. J. Gerontol. Ser. A Biol. Sci. Med. Sci..

[10] Richards J.B., Yuan X., Geller F., Waterworth D., Bataille V., Glass D., Song K., Waeber G., Vollenweider P., Aben K.K.H. (2008). Male-Pattern Baldness Susceptibility Locus at 20p11. Nat. Genet..

[11] Pirastu N., Joshi P.K., De Vries P.S., Cornelis M.C., McKeigue P.M., Keum N., Franceschini N., Colombo M., Giovannucci E.L., Spiliopoulou A. (2017). GWAS for Male-Pattern Baldness Identifies 71 Susceptibility Loci Explaining 38% of the Risk. Nat. Commun..

[12] Heilmann-Heimbach S., Herold C., Hochfeld L.M., Hillmer A.M., Nyholt D.R., Hecker J., Javed A., Chew E.G.Y., Pechlivanis S., Drichel D. (2017). Meta-Analysis Identifies Novel Risk Loci and Yields Systematic Insights into the Biology of Male-Pattern Baldness. Nat. Commun..

[13] Kim I.Y., Kim J.H., Choi J.E., Yu S.J., Kim J.H., Kim S.R., Choi M.S., Kim M.H., Hong K.W., Park B.C. (2022). The First Broad Replication Study of SNPs and a Pilot Genome-Wide Association Study for Androgenetic Alopecia in Asian Populations. J. Cosmet. Dermatol..

[14] Li Y., Huang H., Liang B., Xiao F.L., Zhou F.S., Zheng X.D., Yang S., Zhang X.J. (2024). Association Study Reveals a Susceptibility Locus with Male Pattern Baldness in the Han Chinese Population. Front. Genet..

[15] Janivara R., Hazra U., Pfennig A., Harlemon M., Kim M.S., Eaaswarkhanth M., Chen W.C., Ogunbiyi A., Kachambwa P., Petersen L.N. (2025). Uncovering the Genetic Architecture and Evolutionary Roots of Androgenetic Alopecia in African Men. Hum. Genet. Genom. Adv..

[16] Hillmer A.M., Flaquer A., Hanneken S., Eigelshoven S., Kortüm A.K., Brockschmidt F.F., Golla A., Metzen C., Thiele H., Kolberg S. (2008). Genome-Wide Scan and Fine-Mapping Linkage Study of Androgenetic Alopecia Reveals a Locus on Chromosome 3q26. Am. J. Hum. Genet..

[17] Heilmann S., Brockschmidt F.F., Hillmer A.M., Hanneken S., Eigelshoven S., Ludwig K.U., Herold C., Mangold E., Becker T., Kruse R. (2013). Evidence for a Polygenic Contribution to Androgenetic Alopecia. Br. J. Dermatol..

[18] Deng Y., Wang M., He Y., Liu F., Chen L., Xiong X. (2023). Cellular Senescence: Ageing and Androgenetic Alopecia. Dermatology.

[19] Choi E., Song J., Lee Y., Jeong Y., Jang W. (2024). Prioritizing Susceptibility Genes for the Prognosis of Male-Pattern Baldness with Transcriptome-Wide Association Study. Hum. Genom..

[20] Henne S.K., Aldisi R., Sivalingam S., Hochfeld L.M., Borisov O., Krawitz P.M., Maj C., Nöthen M.M., Heilmann-Heimbach S. (2023). Analysis of 72,469 UK Biobank Exomes Links Rare Variants to Male-Pattern Hair Loss. Nat. Commun..

[21] Ellis J.A., Stebbing M., Harrap S.B. (2001). Polymorphism of the Androgen Receptor Gene Is Associated with Male Pattern Baldness. J. Investig. Dermol..

[22] Hillmer A.M., Hanneken S., Ritzmann S., Becker T., Freudenberg J., Brockschmidt F.F., Flaquer A., Freudenberg-Hua Y., Jamra R.A., Metzen C. (2005). Genetic Variation in the Human Androgen Receptor Gene Is the Major Determinant of Common Early-Onset Androgenetic Alopecia. Am. J. Hum. Genet..

[23] Marcińska M., Pośpiech E., Abidi S., Andersen J.D., Van Den Berge M., Carracedo Á., Eduardoff M., Marczakiewicz-Lustig A., Morling N., Sijen T. (2015). Evaluation of DNA Variants Associated with Androgenetic Alopecia and Their Potential to Predict Male Pattern Baldness. PLoS ONE.

[24] Heilmann S., Kiefer A.K., Fricker N., Drichel D., Hillmer A.M., Herold C., Tung J.Y., Eriksson N., Redler S., Betz R.C. (2013). Androgenetic Alopecia: Identification of Four Genetic Risk Loci and Evidence for the Contribution of WNT Signaling to Its Etiology. J. Investig. Dermatol..

[25] Charoensuksira S., Surinlert P., Krajarng A., Nualsanit T., Payuhakrit W., Panpinyaporn P., Khumsri W., Thanasarnaksorn W., Suwanchinda A., Hongeng S. (2025). Progenitor Cell Dynamics in Androgenetic Alopecia: Insights from Spatially Resolved Transcriptomics. Int. J. Mol. Sci..

[26] Liu Q., Tang Y., Huang Y., Wang J., Yang K., Zhang Y., Pu W., Liu J., Shi X., Ma Y. (2022). Insights into Male Androgenetic Alopecia Using Comparative Transcriptome Profiling: Hypoxia-Inducible Factor-1 and Wnt/β-Catenin Signalling Pathways. Br. J. Dermatol..

[27] Hibino T., Nishiyama T. (2004). Role of TGF-Β2 in the Human Hair Cycle. J. Dermatol. Sci..

[28] Brockschmidt F.F., Heilmann S., Ellis J.A., Eigelshoven S., Hanneken S., Herold C., Moebus S., Alblas M.A., Lippke B., Kluck N. (2011). Susceptibility Variants on Chromosome 7p21.1 Suggest HDAC9 as a New Candidate Gene for Male-Pattern Baldness. Br. J. Dermatol..

[29] Zhuo F.L., Xu W., Wang L., Wu Y., Xu Z.L., Zhao J.Y. (2012). Androgen Receptor Gene Polymorphisms and Risk for Androgenetic Alopecia: A Meta-Analysis. Clin. Exp. Dermatol..

[30] Ambra R., Mastroeni S., Manca S., Mannooranparampil T.J., Virgili F., Marzani B., Pinto D., Fortes C. (2025). Genetic Variants and Lifestyle Factors in Androgenetic Alopecia Patients: A Case–Control Study of Single Nucleotide Polymorphisms and Their Contribution to Baldness Risk. Nutrients.

[31] Li R., Brockschmidt F.F., Kiefer A.K., Stefansson H., Nyholt D.R., Song K., Vermeulen S.H., Kanoni S., Glass D., Medland S.E. (2012). Six Novel Susceptibility Loci for Early-Onset Androgenetic Alopecia and Their Unexpected Association with Common Diseases. PLoS Genet..

[32] Lee J., Choi J.E., Ha J., Kim Y., Lee C., Hong K.W. (2024). Genetic Differences between Male and Female Pattern Hair Loss in a Korean Population. Life.

[33] Francès M.P., Vila-Vecilla L., Russo V., Caetano Polonini H., de Souza G.T. (2024). Utilising SNP Association Analysis as a Prospective Approach for Personalising Androgenetic Alopecia Treatment. Dermatol. Ther..

[34] Redler S., Messenger A.G., Betz R.C. (2017). Genetics and Other Factors in the Aetiology of Female Pattern Hair Loss. Exp. Dermatol..

[35] Redler S., Brockschmidt F.F., Tazi-Ahnini R., Drichel D., Birch M.P., Dobson K., Giehl K.A., Herms S., Refke M., Kluck N. (2012). Investigation of the Male Pattern Baldness Major Genetic Susceptibility Loci AR/EDA2R and 20p11 in Female Pattern Hair Loss. Br. J. Dermatol..

[36] Herskovitz I., Tosti A. (2013). Female Pattern Hair Loss. Int. J. Endocrinol. Metab..

[37] Daunton A., Harries M., Sinclair R., Paus R., Tosti A., Messenger A. (2023). Chronic Telogen Effluvium: Is It a Distinct Condition? A Systematic Review. Am. J. Clin. Dermatol..

[38] Nuwaihyd R., Redler S., Heilmann S., Drichel D., Wolf S., Birch P., Dobson K., Lutz G., Giehl K.A., Kruse R. (2014). Investigation of Four Novel Male Androgenetic Alopecia Susceptibility Loci: No Association with Female Pattern Hair Loss. Arch. Dermatol. Res..

[39] Ho C.Y., Chen J.Y.F., Hsu W.L., Yu S., Chen W.C., Chiu S.H., Yang H.R., Lin S.Y., Wu C.Y. (2023). Female Pattern Hair Loss: An Overview with Focus on the Genetics. Genes.

[40] Ha S.-J., Kim J.-S., Myung J.-W., Lee H.-J., Kim J.-W. (2003). Analysis of Genetic Polymorphisms of Steroid 5a-Reductase Type 1 and 2 Genes in Korean Men with Androgenetic Alopecia. J. Dermatol. Sci..

[41] Liang B., Yang C., Zuo X., Li Y., Ding Y., Sheng Y., Zhou F., Cheng H., Zheng X., Chen G. (2013). Genetic Variants at 20p11 Confer Risk to Androgenetic Alopecia in the Chinese Han Population. PLoS ONE.

[42] Lee W.S., Lee H.J. (2012). Characteristics of Androgenetic Alopecia in Asian. Ann. Dermatol..

[43] Otberg N., Finner A.M., Shapiro J. (2007). Androgenetic Alopecia. Endocrinol. Metab. Clin. N. Am..

[44] Henne S.K., Nöthen M.M., Heilmann-Heimbach S. (2023). Male-Pattern Hair Loss: Comprehensive Identification of the Associated Genes as a Basis for Understanding Pathophysiology. Med. Genet..

[45] Qi T., Song L., Guo Y., Chen C., Yang J. (2024). From Genetic Associations to Genes: Methods, Applications, and Challenges. Trends Genet..

[46] Gay N.R., Gloudemans M., Antonio M.L., Abell N.S., Balliu B., Park Y., Martin A.R., Musharoff S., Rao A.S., Aguet F. (2020). Impact of Admixture and Ancestry on EQTL Analysis and GWAS Colocalization in GTEx. Genome Biol..

[47] Lu Z., Gopalan S., Yuan D., Conti D.V., Pasaniuc B., Gusev A., Mancuso N. (2022). Multi-Ancestry Fine-Mapping Improves Precision to Identify Causal Genes in Transcriptome-Wide Association Studies. Am. J. Hum. Genet..

[48] Wu S., Yu Y., Liu C., Zhang X., Zhu P., Peng Y., Yan X., Li Y., Hua P., Li Q. (2022). Single-Cell Transcriptomics Reveals Lineage Trajectory of Human Scalp Hair Follicle and Informs Mechanisms of Hair Graying. Cell Discov..

[49] Takahashi R., Grzenda A., Allison T.F., Rawnsley J., Balin S.J., Sabri S., Plath K., Lowry W.E. (2020). Defining Transcriptional Signatures of Human Hair Follicle Cell States. J. Investig. Dermatol..

[50] Shim J., Park J., Abudureyimu G., Kim M.H., Shim J.S., Jang K.T., Kwon E.J., Jang H.S., Yeo E., Lee J.H. (2022). Comparative Spatial Transcriptomic and Single-Cell Analyses of Human Nail Units and Hair Follicles Show Transcriptional Similarities between the Onychodermis and Follicular Dermal Papilla. J. Investig. Dermatol..

[51] Ober-Reynolds B., Wang C., Ko J.M., Rios E.J., Aasi S.Z., Davis M.M., Oro A.E., Greenleaf W.J. (2023). Integrated Single-Cell Chromatin and Transcriptomic Analyses of Human Scalp Identify Gene-Regulatory Programs and Critical Cell Types for Hair and Skin Diseases. Nat. Genet..

[52] Mehta A., Motavaf M., Raza D., McLure A.J., Osei-Opare K.D., Bordone L.A., Gru A.A. (2025). Revolutionary Approaches to Hair Regrowth: Follicle Neogenesis, Wnt/ß-Catenin Signaling, and Emerging Therapies. Cells.

[53] Wen L., Fan Z., Huang W., Miao Y., Zhang J., Liu B., Zhu D., Dai D., Zhang J., Le D. (2025). Retinoic Acid Drives Hair Follicle Stem Cell Activation via Wnt/β-Catenin Signalling in Androgenetic Alopecia. J. Eur. Acad. Dermatol. Venereol..

[54] Ntshingila S., Oputu O., Arowolo A.T., Khumalo N.P. (2023). Androgenetic Alopecia: An Update. JAAD Int..

[55] Hayes V.M., Severi G., Padilla E.J.D., Morris H.A., Tilley W.D., Southey M.C., English D.R., Sutherland R.L., Hopper J.L., Boyle P. (2007). 5α-Reductase Type 2 Gene Variant Associations with Prostate Cancer Risk, Circulating Hormone Levels and Androgenetic Alopecia. Int. J. Cancer.

[56] Zhou Z., Song S., Gao Z., Wu J., Ma J., Cui Y. (2019). The Efficacy and Safety of Dutasteride Compared with Finasteride in Treating Men with Androgenetic Alopecia: A Systematic Review and Meta-Analysis. Clin. Interv. Aging.

[57] Hu A.C., Chapman L.W., Mesinkovska N.A. (2019). The Efficacy and Use of Finasteride in Women: A Systematic Review. Int. J. Dermatol..

[58] Messenger A., Rundegren J. (2004). Minoxidil: Mechanisms of Action on Hair Growth. Br. J. Dermatol..

[59] Goren A., Castano J.A., McCoy J., Bermudez F., Lotti T. (2014). Novel Enzymatic Assay Predicts Minoxidil Response in the Treatment of Androgenetic Alopecia. Dermatol. Ther..

[60] Jimenez-Cauhe J., Vaño-Galvan S., Mehta N., Hermosa-Gelbard A., Ortega-Quijano D., Buendia-Castaño D., Fernández-Nieto D., Porriño-Bustamante M., Saceda-Corralo D., Pindado-Ortega C. (2024). Hair Follicle Sulfotransferase Activity and Effectiveness of Oral Minoxidil in Androgenetic Alopecia. J. Cosmet. Dermatol..

[61] Gaboardi H., Russo V., Vila-Vecilla L., Patel V., De Souza G.T. (2025). 26-SNP Panel Aids Guiding Androgenetic Alopecia Therapy and Provides Insight into Mechanisms of Action. Cosmetics.

[62] Cecchin E., De Mattia E., Mazzon G., Cauci S., Trombetta C., Toffoli G. (2014). A Pharmacogenetic Survey of Androgen Receptor (CAG)n and (GGN)n Polymorphisms in Patients Experiencing Long Term Side Effects after Finasteride Discontinuation. Int. J. Biol. Markers.

[63] Cauci S., Chiriacò G., Cecchin E., Toffoli G., Xodo S., Stinco G., Trombetta C. (2017). Androgen Receptor (AR) Gene (CAG)n and (GGN)n Length Polymorphisms and Symptoms in Young Males with Long-Lasting Adverse Effects After Finasteride Use Against Androgenic Alopecia. Sex. Med..

[64] Martinez-Chapoy D., Cruz-Arroyo F.J., Ancer-Leal F.D., Rodriguez-Leal R.A., Camacho-Zamora B.D., Guzman-Sanchez D.A., Espinoza-Gonzalez N.A., Martinez-Jacobo L., Marino-Martinez I.A. (2022). Pilot Study: Genetic Distribution of AR, FGF5, SULT1A1 and CYP3A5 Polymorphisms in Male Mexican Population with Androgenetic Alopecia. Int. J. Mol. Epidemiol. Genet..

[65] Faghihi G., Iraji F., Siadat A.H., Saber M., Jelvan M., Hoseyni M.S. (2022). Comparison between “5% Minoxidil plus 2% Flutamide” Solution vs. “5% Minoxidil” Solution in the Treatment of Androgenetic Alopecia. J. Cosmet. Dermatol..

[66] Aleissa M. (2023). The Efficacy and Safety of Oral Spironolactone in the Treatment of Female Pattern Hair Loss: A Systematic Review and Meta-Analysis. Cureus.

[67] Seyed Jafari S.M., Heidemeyer K., Hunger R.E., de Viragh P.A. (2024). Safety of Antiandrogens for the Treatment of Female Androgenetic Alopecia with Respect to Gynecologic Malignancies. J. Clin. Med..

[68] Donnelly C., Minty I., Dsouza A., Wong Y.Y., Mukhopadhyay I., Nagarajan V., Rupra R., Charles W.N., Khajuria A. (2024). The Role of Platelet-Rich Plasma in Androgenetic Alopecia: A Systematic Review. J. Cosmet. Dermatol..

[69] Perez S.M., Vattigunta M., Kelly C., Eber A. (2025). Low-Level Laser and LED Therapy in Alopecia: A Systematic Review and Meta-Analysis. Dermatol. Surg..

[70] Perez S.M., AlSalman S.A., Nguyen B., Tosti A. (2025). Botulinum Toxin in the Treatment of Hair and Scalp Disorders: Current Evidence and Clinical Applications. Toxins.

[71] Queen D., Avram M.R. (2025). Hair Transplantation: State of the Art. Dermatol. Surg..

[72] Dhurat R., Daruwalla S., Pai S., Kovacevic M., McCoy J., Shapiro J., Sinclair R., Vano-Galvan S., Goren A. (2022). SULT1A1 (Minoxidil Sulfotransferase) Enzyme Booster Significantly Improves Response to Topical Minoxidil for Hair Regrowth. J. Cosmet. Dermatol..

[73] Roberts J., Desai N., McCoy J., Goren A. (2014). Sulfotransferase Activity in Plucked Hair Follicles Predicts Response to Topical Minoxidil in the Treatment of Female Androgenetic Alopecia. Dermatol. Ther..

[74] Li Y., Dong T., Wan S., Xiong R., Jin S., Dai Y., Guan C. (2024). Application of Multi-Omics Techniques to Androgenetic Alopecia: Current Status and Perspectives. Comput. Struct. Biotechnol. J..

[75] Pozo-Pérez L., Tornero-Esteban P., López-Bran E. (2024). Clinical and Preclinical Approach in AGA Treatment: A Review of Current and New Therapies in the Regenerative Field. Stem. Cell Res. Ther..

[76] Pietrauszka K., Bergler-Czop B. (2022). Sulfotransferase SULT1A1 Activity in Hair Follicle, a Prognostic Marker of Response to the Minoxidil Treatment in Patients with Androgenetic Alopecia: A Review. Postepy Dermatol. Alergol..

[77] Rossi A., Caro G. (2024). Efficacy of the Association of Topical Minoxidil and Topical Finasteride Compared to Their Use in Monotherapy in Men with Androgenetic Alopecia: A Prospective, Randomized, Controlled, Assessor Blinded, 3-Arm, Pilot Trial. J. Cosmet. Dermatol..

[78] Suchonwanit P., Iamsumang W., Rojhirunsakool S. (2019). Efficacy of Topical Combination of 0.25% Finasteride and 3% Minoxidil Versus 3% Minoxidil Solution in Female Pattern Hair Loss: A Randomized, Double-Blind, Controlled Study. Am. J. Clin. Dermatol..

[79] Chen L., Zhang J., Wang L., Wang H., Chen B. (2020). The Efficacy and Safety of Finasteride Combined with Topical Minoxidil for Androgenetic Alopecia: A Systematic Review and Meta-Analysis. Aesthetic Plast. Surg..

